# Optimisation of Clutch Disc Friction Material Using a Multi-Layer Perceptron Artificial Neural Network

**DOI:** 10.3390/polym16243588

**Published:** 2024-12-22

**Authors:** George Bălășoiu, Cristian Munteniță, Valentin Tiberiu Amortila, Larisa Titire

**Affiliations:** Mechanical Engineering Department, Dunarea de Jos University of Galati, 800201 Galati, Romania; cristian.muntenita@ugal.ro (C.M.); valentin.amortila@ugal.ro (V.T.A.); larisa.titire@ugal.ro (L.T.)

**Keywords:** clutch disc, friction materials, artificial neural network, pin-on-disc test, material optimisation

## Abstract

This paper presents an analysis of four clutch disc friction materials (from different manufacturers) used in manual transmissions. Scanning electron microscopy and energy-dispersive X-ray spectroscopy were employed for the microstructural and chemical characterisation of the friction materials. To reveal the tribological properties of the selected clutch discs, three measurements of the friction coefficient between the material and the cast iron disc were conducted. The findings were employed to construct an artificial neural network using Easy NN software (V 14), with the objective of optimising the friction material. The chemical composition of the friction materials was employed as the input data, whereas the minimum, maximum, and average values of the friction coefficient, as well as the temperature generated during friction, were utilised as the output data. To assess the efficacy of the neural network, the correlation between the importance of input data and their sensitivity to output data was examined. It was determined that the model with three hidden layers exhibited a notable correlation between the six most influential chemical elements and their sensitivity. Based on this neural model, the chemical composition of the friction disc materials was optimised using the “Query” mode, aiming to minimise discrepancies in friction coefficients and temperature development.

## 1. Introduction

Dry clutch friction discs are typically utilised within the manual transmission, which represents the most common transmission type at the European level [1]. The clutch disc plays a significant role in ensuring the efficient operation of the transmission system in a vehicle. The primary function of a clutch is to provide a temporary disconnection between the engine’s main shaft and the transmission system. This disconnection allows the vehicle to start moving, to change gears, or make stops without interruption of the engine’s operation. At the same time, a temporary separation is necessary for maintaining control of the vehicle and preventing damage to transmission system components during operation [1,2,3]. Nowadays, technical developments in automotive technology have led to the use of clutch discs in combination with automatic transmissions [4]. In the case of the automatic transmissions, clutch discs are suitable for use in a variety of environments, including dry and wet conditions. The main advantages of dry clutches are fuel economy, low maintenance and production costs, and high reliability [1].

**Figure 1:**
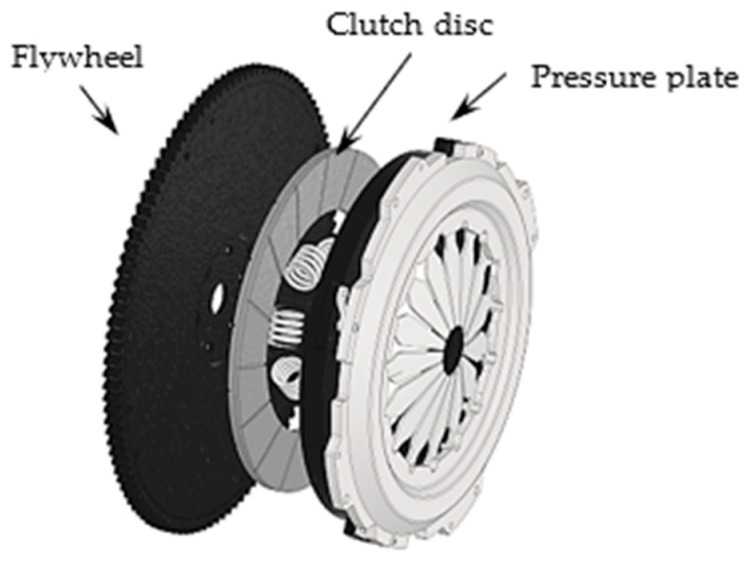
General structure of a manual transmission dry clutch.

Figure 1 shows the general architecture of a dry clutch assembly used in manual transmissions. The main elements that can be identified are the flywheel, clutch disc, and pressure plate. When the clutch pedal is depressed, the flywheel and pressure plate (which are connected by bolts) rotate with the engine speed, while the clutch disc (which is connected to the transmission main shaft) is stationary. When the vehicle starts moving, the pressure plate tightens the clutch disc on the flywheel. The angular speeds of these components are different and in order to synchronise them, sliding friction (clutch slipping) is required between the clutch disc and the two surfaces: the flywheel and the pressure plate [5].

This sliding friction occurs during the shifting process, as well as during vehicle acceleration. In the second case, the most intense slipping process is recorded, leading to a progressive thinning of the active material layer of the friction disc and high frictional heat generation [3,6,7]. Friction discs are subject to continuous wear during normal clutch operation due to the constant frictional contact between the disc surface and the pressure plate or flywheel. Multiple defects such as deformation, cracking, and/or vibration will occur due to the excessive temperature rise. As a result, the clutch may fail prematurely [6,7].

The literature presents many studies in this field that treat the thermal behaviour of the friction material used in clutch disc production. For example, Czél et al. [8] carried out a numerical analysis of a dry clutch model with ceramic material using the finite element method and then compared the results with experimental data. The study was conducted under the hypothesis that temporal and spatial variations influence the convection coefficient and the heat partition coefficient. The results showed a strong correlation with the experimentally measured temperatures. Al-Zubaidi et al. [9] proposed an experimental method to evaluate the thermal response of friction materials in a dry clutch by studying how variations in impact load and ambient temperature affect the performance of the clutch. They found that the coefficient of friction and frictional force are significantly affected by ambient temperature and load intensity, especially at higher levels.

For those reasons, the chemical composition of the friction material is important since it plays an important role in the temperature dissipation [6]. Selection of the friction material is essential because it must comply with certain conditions imposed, such as high values of the coefficient of friction and its maintenance in a wide range of temperatures, high thermal conductivity, high resistance under corrosion and mechanical stress (scoring, galling, shear force, mechanical damage, etc.), and environmental friendliness [10,11].

## 2. Materials and Methods

### 2.1. Materials

In general, friction materials for clutch discs are made from materials with superior tribological properties that can withstand the intense friction and mechanical wear caused by repeated contact with other system components. For example, according to the literature, two types of composite materials are typically used for friction discs in wet clutches: paper- and copper-based friction materials [12].

The paper-based friction material has a chemical composition based on four main categories (similar to brake pad friction material) [13]: friction modifiers (as carbon particles), reinforcing fibres (aramid fibre, ceramic or carbon fibre, etc.), binders (commonly used are phenolic resin and modified phenolic resin), and fillers (diatomaceous earth containing silica and alumina minerals) [12,14,15,16].

Due to their great mechanical and tribological proprieties, the copper-based friction materials are commonly used for wet clutches of heavy vehicles. Usually, they are manufactured by powder metallurgy [12].

A fundamental parameter affecting the efficient operation of friction discs is the coefficient of friction. An appropriate coefficient of friction is critical for efficient torque transfer between the engine and the transmission system, minimising energy loss due to excessive slippage. It also provides effective adhesion between the friction disc and the pressure plate or flywheel, preventing overheating and accelerated wear. These factors contribute to an extended clutch life and reliable operation. These materials are selected for their ability to provide an adequate coefficient of friction, thermal stability, and resistance to mechanical stress. In addition, the surface of the friction discs is designed to provide optimum contact with the pressure plate or flywheel, often with grooves or slots to improve friction and dissipate heat generated during operation [14,17].

**Figure 2:**
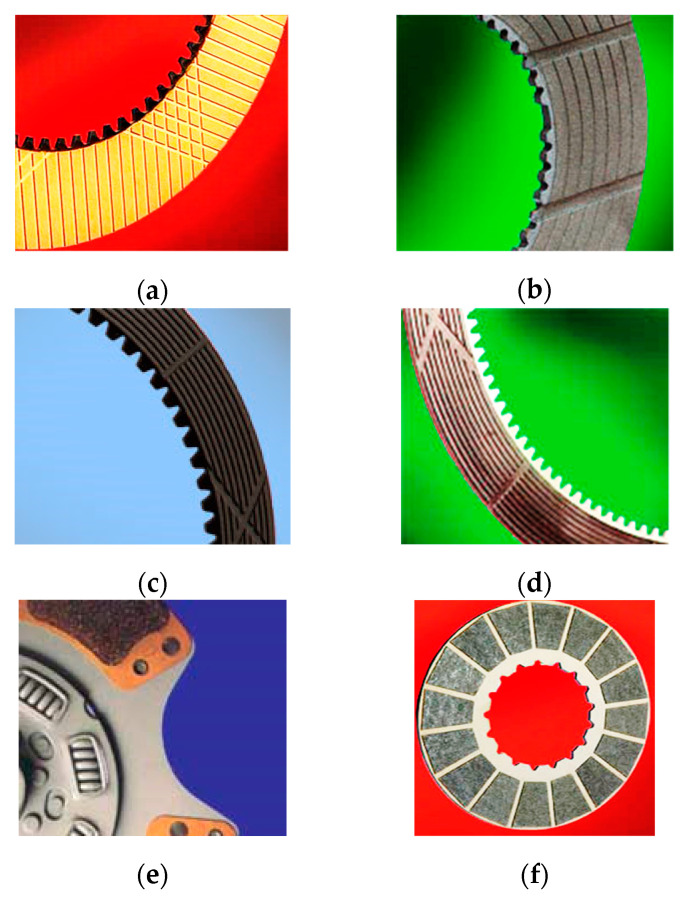
Types of friction discs: (**a**) paper-based friction material; (**b**) disc with molybdenum friction layer; (**c**) disc with graphite friction layer; (**d**) disc with elastomer friction layer; (**e**) disc with ceramic friction layer; (**f**) disc with carbon friction layer.

Figure 2 shows some design solutions for clutch disc surfaces.

These design features help better manage the kinetic energy transferred during shifting, reducing the risk of overheating and component degradation. In this way, friction discs ensure efficient and safe power transfer between the engine and transmission system, with a balance between durability, performance, and vehicle control. The performance of the friction materials and their surface design are important to the reliable operation of the clutch.

The quality of the clutch friction plates has a direct effect on clutch performance. Lower-quality friction plates or those showing advanced wear can cause undesirable effects such as excessive clutch slipping, difficulty shifting, or in extreme cases, premature failure of the entire clutch system.

The coefficient of friction of the clutch discs is influenced by several factors that determine the performance and durability of the clutch system:

(i) Chemical components that are used in the manufacture of the clutch disc friction material. According to the literature, the chemical composition of clutch friction materials plays an important role in tribological behaviour [18]. For example, Wongpayakyotin et al. [19] and Bijwe et al. [20] stated that the binders can modify wear properties to the clutch friction materials. Furthermore, according to Kumar et al. [21], copper can improve the proprieties of friction materials.

(ii) The surface condition of the friction material of the clutch disc (smooth surfaces tend to have a lower coefficient of friction, while greater roughness can increase adhesion but accelerate material wear). Surface roughness is a critical factor in the performance of tribological systems. Research conducted by Kubiak et al. [22] demonstrated that the initial surface roughness significantly affects the friction coefficient during the transition from partial to full sliding. Similarly, Fernandes et al. [23] investigated how varying the surface roughness of cast discs influences the wear rate and coefficient of friction in clutch systems. Their findings revealed that when using a cast iron disc with a smoother surface, the wear rate significantly decreased, and the coefficient of friction recorded a stable and high value during the running-in period. This outcome was attributed to the maintaining of tribofilms and the reduction in stress concentrations in the contact area under high tribological loads.

(iii) The force applied by the pressure plate against the friction disc and the flywheel. A higher contact pressure leads to an increase in the coefficient of friction values, and at the same time, excessive pressure is unwanted;

(iv) The temperature of the friction material surface is a determining factor in the variation in the coefficient of friction values. The friction coefficient tends to decrease with temperature, which can negatively affect the performance of the clutch, reducing the efficiency of torque transfer and increasing the risk of excessive slipping. High temperatures can accelerate the wear of friction discs, leading to their premature damage. In the case of improper use or overuse of the clutch, overheating can cause deformation, cracking, or even melting of the disc material, seriously affecting the reliability of the clutch system.

In this study, four different types of dry clutch disc friction materials from different manufacturers were considered. Due to confidentiality reasons, the chemical composition of the friction materials is not disclosed by the manufacturers, which is their property. Therefore, scanning electron microscopy and energy dispersive X-ray spectroscopy analysis were used for microstructural and chemical characterisation. The samples are referred to here as disc 1, disc 2, disc 3, and disc 4 to avoid disclosure of the manufacturer’s trade name. The SEM and EDX analysis were performed using a microscope provided by Oxford Instruments, and the AZtec software was utilised for data acquisition and analysis. AZtec offers significant advantages, including precise elemental mapping, advanced imaging capabilities, and high-resolution data collection at both micro- and nanoscale levels, ensuring reliable and reproducible results.

For the tribological tests, we used a flat pin with a diameter of 10 mm from each type of clutch disc friction material, assuming that a contact area of 78.55 mm^2^ is enough for a global characterisation. As a counter piece, we used a grey cast iron disc with a diameter of 260 mm.

### 2.2. Tribological Tests

The tribological tests were conducted by simulating the conditions of maximum clutch stress, which occur during the departure process, characterised by the longest slipping interval of the clutch. In normal conditions, this process can take up to 5–7 s.

The coefficient of friction of clutch disc friction materials can be determined by several experimental methods and testing techniques. According to [18], for tribological investigation of the friction materials, a pin-on-disc tribometer is generally used. Therefore, a test rig with a pin-on-disc configuration was used. The normal force was applied with a dead weight of 50 N. The drag test was conducted for 4.6 s from a stand-still position of the disc to 1500 rpm. For each type of clutch disc friction material, we conducted three tests. During the tests, parameters such as disc surface temperature and coefficient of friction were recorded. For each test, the surface temperature of the friction material was identical. The tests were conducted at an ambient temperature of 22 ± 1 °C.

**Figure 3:**
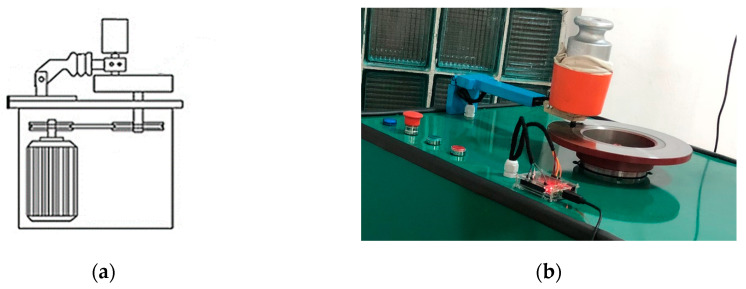
Pin-on-disc configuration: (**a**) schematic representation of the pin-on-disc configuration; (**b**) test rig with pin-on-disc configuration.

Figure 3a shows a schematic representation of the pin-on-disc configuration, while Figure 3b presents the test rig configuration. As Figure 3b shows, the coefficient of friction value was calculated as the ratio between the shear force and normal force. The shear force was recorded with a force cell while the normal force was applied with a dead weight. The temperature of the disc surface was recorded with an infrared temperature sensor with a deviation of 0.02 °C.

### 2.3. Artificial Neural Network Analysis

Based on the results of the chemical analysis and tribological tests, an artificial neural network (ANN) analysis was proposed. ANNs are based on mathematical models inspired by the structure and functioning of the human brain, and they are used in artificial intelligence to learn and perform specific tasks [24]. The structure of an ANN consists of elementary units (artificial neurons) interconnected by connections (synapses, axons) weighted with numerical values (weights). Depending on the direction of information flow in the network, there are two main categories of ANN: feed-forward networks, where information flows from input to output, and recurrent networks, where information circulates in both directions [25]. In situations where the goal is to predict or analyse phenomena, feed-forward structures are recommended [26].

The optimisation process of the friction material used in clutch discs was carried out using EasyNN software (V 14) (an artificial neural network-based tool) and involved the following steps: data preparation, EasyNN modelling, model training, optimisation, results analysis, and implementation [27]. The data were divided into training, validation, and test sets (70%–15%–15%) during the data preparation phase.

**Figure 4:**
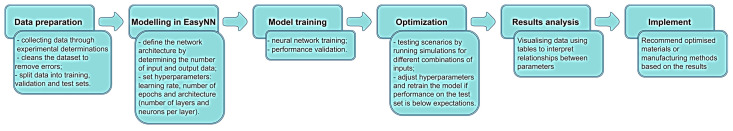
Optimisation process using ANN.

Figure 4 shows how the optimisation process was carried out.

One of the most widely used ANN architectures is the multi-layer perceptron (MLP), which is characterised by a structure in which information flows unidirectionally from the input layer to the output layer (feed-forward) [8]. In this study, the EasyNN software (V 14) was used, which implements a feed-forward architecture (unidirectional flow of information, from input to output). This allows the generation of neural networks with one, two, or three hidden layers. For the ANN training, the chemical composition of the four clutch disc friction materials considered in this study was used as input data, while the results obtained after the tribological tests were used as output data.

## 3. Results and Discussion

### 3.1. Chemical Characterisation

The chemical composition of the disc friction material is not provided for confidentiality reasons. In this regard, the four types of clutch disc friction materials considered in this study were analysed using EDX analysis.

**Figure 5:**
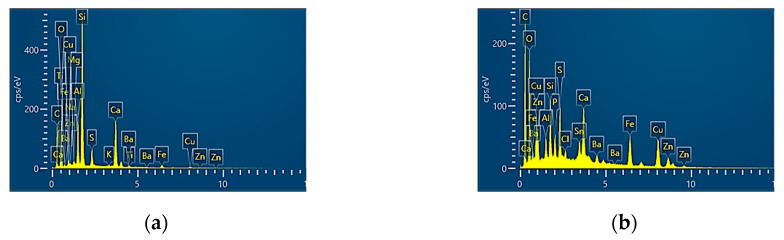

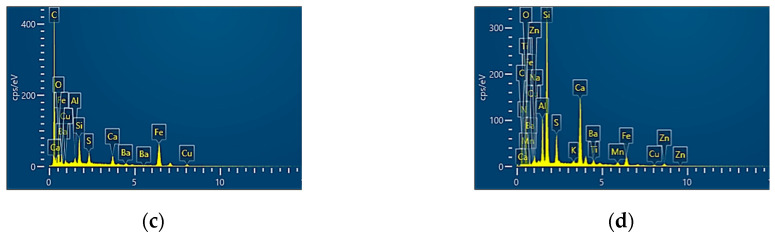
EDX analysis of the clutch disc friction materials: (**a**) disc 1; (**b**) disc 2; (**c**) disc 3; (**d**) disc 4.

Figure 5 graphically shows the chemical composition of one of the most representative zones from the friction material surfaces. It can clearly be seen that the main chemical components found, in the case of all clutch disc friction materials, in high concentrations are carbon (C) and oxygen (O), followed by aluminium (Al), silicon (Si), sulphur (S), calcium (Ca), iron (Fe), cooper (Cu), and barium (Ba), but in small quantities.

It can be observed that each manufacturer has a specific chemical composition, which is highlighted by additional elements’ introduction.

According to the literature, the high concentration of C in the friction materials occurs due to the presence of graphite (a solid lubricant used in general to reduce the wear rate), but it may also come from phenolic resin (used as binder) [18,28,29]. The presence of Cu indicates that the friction materials contain brass. Generally, brass is used as brass fibre (soft reinforcing fibres) or as brass powder (used as filler) [28,30,31]. Ca and Si with O form calcium silicate (Ca_2_SiO_4_), which usually is used to increase the stability of the coefficient of friction [32]. To increase the values of the coefficient of friction and also reduce the wear rate, according to [33,34], we used Ba.

### 3.2. Tribological Characterisation

**Figure 6:**
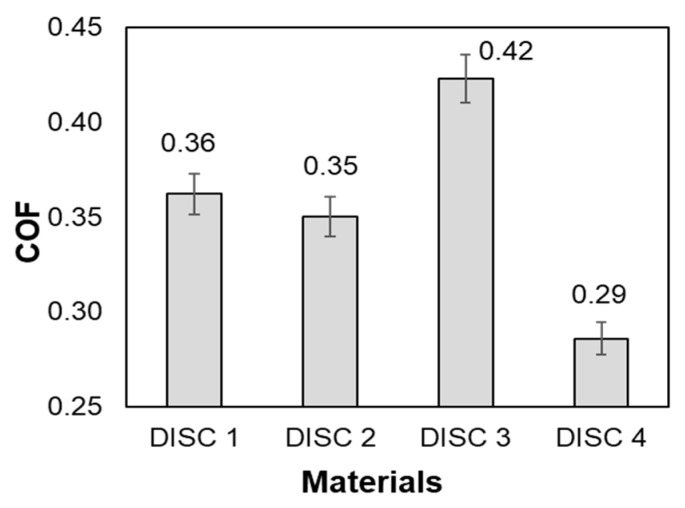
Mean COF values as function of clutch disc friction material.

The mean values of the coefficient of friction of the four clutch friction materials considered in this study are shown in Figure 6. It can be observed that the highest coefficient of friction value (0.423) was obtained in the case of the disc 3 friction material, while the lowest coefficient of friction value (0.286) was obtained in the case of the disc 4 friction material.

**Figure 7:**
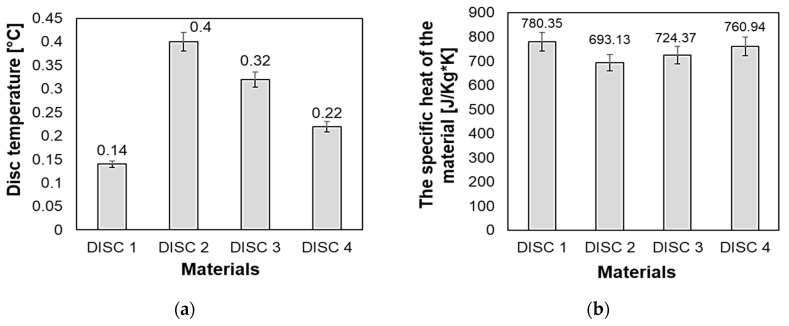
(**a**) Disc surface temperature variation as a function of disc friction material; (**b**) specific heats of the friction materials.

Figure 7a presents the variation in the disc surface temperature from its value at the beginning of the test to its value after the test, while Figure 7b shows the calculated specific heat of friction materials considered in this study.

The specific heat of the friction material was determined using a calculation formula. Specifically, we employed the weighted average method based on the chemical composition of the material. The formula used is as follows:

(1) $$c=\overset{n}{\underset{i=1}{\sum }}\frac{m_{i}\cdot c_{i}}{100}$$

where:

- c represents the specific heat of the composite material;
- *m_i_* represents the mass in the chemical compound of the composite expressed in [wt%];
- *c_i_* represents the specific heat of the chemical compound expressed in [J/kg·K].

From Figure 7, it can be observed that the highest temperature variation was obtained in the case of the disc 2 friction material followed by the disc 3 and disc 4 friction materials. The lowest temperature variation was obtained in the case of disc 1 friction material. It is interesting that the calculated specific heat has values inversely proportional to the temperature variation. The explanation for this trend is that the friction material with a lower specific heat (disc 2 = 693,133 J/Kg·K) heats up faster (T = 0.4 °C) and it requires a smaller amount of energy to increase the temperature by a degree. Thus, it can be concluded that the chemical composition of the friction discs significantly influences their heating rate. When studying the values of the coefficient of friction for the four friction discs (Figure 6), it was not possible to establish a correlation between the increase in temperature and the variation in the coefficient of friction.

### 3.3. Morphological Characterisation

**Figure 8:**
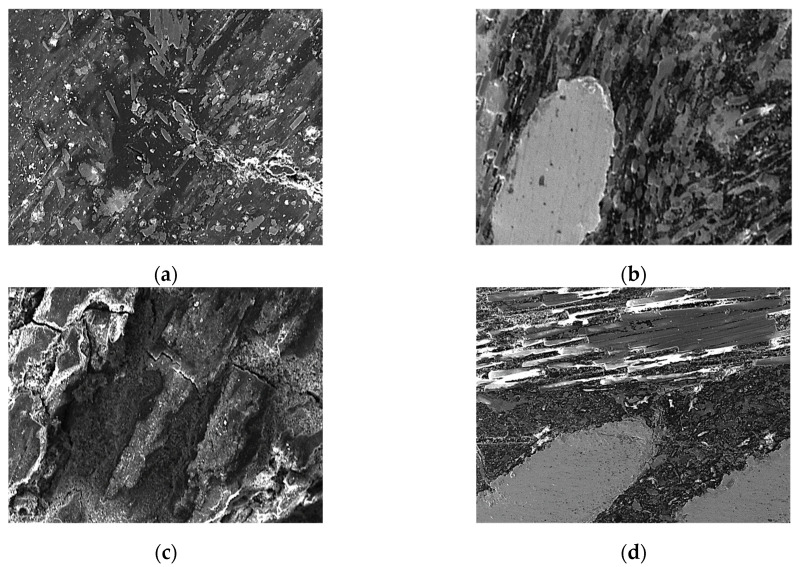
SEM analysis of the clutch disc friction material contact surfaces after the tests: (**a**) disc 1; (**b**) disc 2; (**c**) disc 3; (**d**) disc 4.

After the tribological tests, the surfaces of the pin samples were analysed using SEM. The microstructure of the tested pin surfaces at a magnification of 250× is presented in Figure 8. Overall, a specific microstructure can be seen of a composite friction material. Particularly, in the case of disc 4 friction material, the reinforcing fibres can be observed.

In all studied cases, we observed that the main wear mechanism is three-body abrasion. According to Eriksson et al. [35,36,37,38], the real contact area is smaller, and the load is concentrated in small areas of the friction material surface. These areas are named plateaus. Two type of plateaus can be found: primary and secondary plateaus. The first one appears in the place where the chemical components have a higher hardness, and the second one forms due to debris that appear in contact. These debris form agglomerations and are compacted at the primary plateau base. Depending on the level of stress applied to the system, these plateaus can be destroyed and restored continuously.

Areas with primary plateaus can be found in the case of disc 2 and disc 4. In the case of disc 3 and disc 1, on the contact surfaces, some cracks can be observed, which may have appeared due to thermal stress.

### 3.4. Artificial Neural Network Development

Based on the analysis of the results obtained from tribological tests and microscopic examinations, it can be concluded that the friction materials used in clutch discs are complex composite materials with multiple chemical constituents. The shape, size, and behaviour of these constituents define the tribological properties of the friction material. In this context, the application of a mathematical model to predict the tribological behaviour of new friction materials or to optimise friction materials is a challenging task. Therefore, artificial neural networks have been applied. In this study, the MLP was selected as the artificial neural network model due to its suitability for handling complex relationships between input and output data, particularly in the context of optimising friction materials based on chemical composition and tribological performance. The MLP network can minimise the error function between the actual and desired outputs [8,39]. In this case, the neurons are composed of three layers: (i) first layer is the input layer where the input values are defined, (ii) the second layer comprises one or more hidden layers and represents the brain of this system, (iii) and the third layer is the output layer and presents the output data of the ANN model [39].

After the ANN training, we obtained neural networks with one, two and three hidden layers. As inputs, we used the percentages of the chemical constituents of the four clutch disc friction materials, and as output data, the minimum, maximum, and average values of coefficient of friction and temperature.

**Figure 9:**
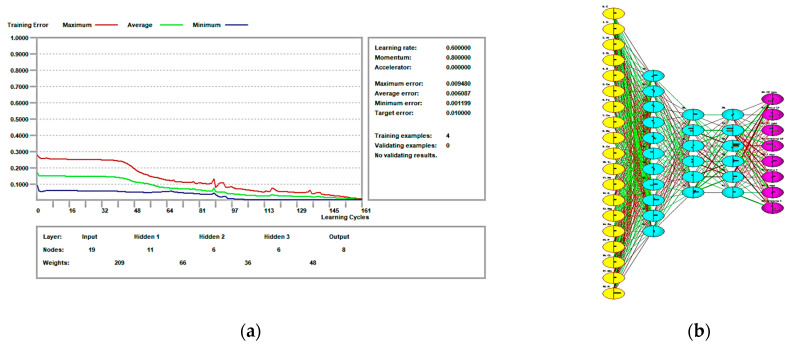
Artificial neural network: (**a**) error evolution in the training stage; (**b**) structure of the ANN model.

Figure 9a presents the error evolution in the training stage of the ANN, while Figure 9b presents the structure of the artificial neural network.

With 161 learning cycles, it can be observed that the average error has a value of approx. 0.006. Also, the ANNs provide the chance of examining the influence of the input data on the output data and the sensitivity of the output data to the input data.

Based on the comparative analysis of different neural network architectures, the configuration that gave the best performance was selected for further optimisation and analysis. The adopted neural network consists of three hidden layers, structured to effectively capture the complex relationships between the input variables (chemical composition percentages) and the output variables (coefficient of friction and temperature). The first hidden layer consists of 11 neurons, providing a robust capacity for initial feature extraction and nonlinear transformation. The second and third hidden layers, each with six neurons, help to refine the learned features and model complex dependencies within the data. The network was trained over 161 learning cycles (epochs), allowing sufficient iterations to minimise error and achieve convergence. A learning rate of 0.6 was used to balance the speed of convergence with the stability of the optimisation process. This configuration demonstrated superior predictive accuracy and a strong ability to generalise the input–output relationships, as evidenced by its performance metrics.

The relative importance parameter, defined as the sum of the absolute values of the input weights, provides an assessment of the influence of an input variable on the output. However, this metric has a clear limitation in that it ignores the potentially significant contribution of weights associated with hidden nodes [40]. According to Senatore et al. [8], the sensitivity index represents the mean variation in the output variable across the full range of a given input variable.

**Figure 10:**
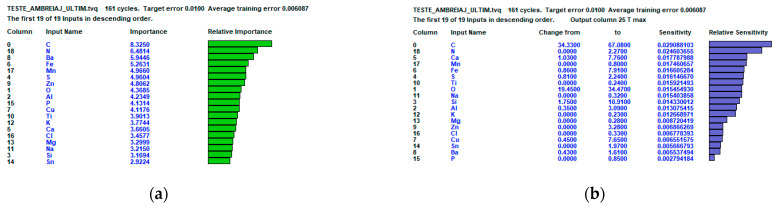
Analysis of the relative importance and relative sensitivity of the artificial neural network: (**a**) analysis of the importance of the input elements for the output data; (**b**) analysis of the sensitivity of the output data to the input data.

Figure 10 illustrates the analysis of the importance of the input elements for the output data, as well as the sensitivity of the output data to the input data.

It is noted that in the case of the three-layer hidden model, five of the first six elements identified in the relative importance are also present in the relative sensitivity. From Figure 10, it can be observed that Cu has the maximum influence on the output data (coefficient of friction and temperature). This agrees with the results obtained in the tribological test, where it can be observed that the friction material of disc 2 (which, according to the EDX analysis, has a significant amount of copper) recorded the highest temperature value.

In the literature, there are some studies that use artificial neural networks to predict the variation in the coefficient of friction, but none of them use as an input dataset the chemical composition of the material [8,39]. Interestingly, the ANN model can also be used to predict output values for input parameters not included in the training dataset (experimentally untested configurations), provided that the new input values fall within the minimum and maximum ranges used during training.

EasyNN offers the “Query” module to vary the values of the input data and predict the values of the output data in real-time. Also, the “Seek High” or “Seek Low” commands can be used to optimise the input data (chemical composition of the clutch disc friction materials) according to a desired value of the output data.

Table 1 shows the chemical compositions of the two friction materials according to the desired COF and temperature values.

As shown in Table 1, the artificial neural network provides two different chemical compositions corresponding to two desired output values: the maximum coefficient of friction (COF) and the minimum temperature variation. The first composition is optimised to achieve the minimum temperature variation, while the second composition is designed to maximise the coefficient of friction. These results demonstrate the ability of the neural network to optimise the chemical composition of friction materials for specific performance objectives, providing tailored solutions for different operational requirements.

## 4. Conclusions

Based on the comparative analysis of four types of clutch disc friction materials from different manufacturers, this study has produced several important conclusions regarding the relationship between chemical composition and tribological performance:

- The use of SEM and EDX techniques revealed significant differences in the chemical compositions of the friction materials. These compositional variations had a direct effect on the performance of the discs, particularly in terms of their coefficient of friction and thermal behaviour during operation. This underscores the critical role of material composition in determining tribological performance.
- Precise measurements of the coefficient of friction between the clutch disc friction materials and the cast iron disc were made. These measurements highlighted the variability in performance of the different materials and provided essential data for the development of the neural network model.
- The data obtained from the experimental measurements were used to construct a neural network model with three hidden layers. This model showed a strong correlation between the input variables (chemical composition) and the output variables (coefficient of friction and temperature). The ability of the model to capture the complex relationships between composition and performance validates its effectiveness as a material optimisation tool.
- The predictive accuracy of the neural network enabled the optimisation of friction material compositions. By minimising discrepancies in friction coefficients and surface temperatures, the model provided insights into achieving an improved and consistent material performance. The model’s “query” capability facilitated this optimisation process.
- The sensitivity analysis revealed a significant consistency between the six most influential constituents and their effect on the friction coefficient and temperature generation. This finding confirms that even small variations in chemical composition can significantly affect the tribological performance of clutch disc materials.
- This study confirmed the importance of optimising material composition to improve tribological performance. The demonstrated relationship between chemical composition and performance provides a basis for the development of advanced friction materials with improved wear resistance and thermal stability.

This research highlights the critical role of chemical composition in the tribological performance of clutch disc friction materials and demonstrates the potential of neural network models as powerful tools for material optimisation. These findings contribute to a deeper understanding of friction materials and their behaviour, paving the way for further advancements in clutch disc technology and material engineering. Future studies should aim to expand the dataset to include friction materials from additional manufacturers and a wider range of operating scenarios to validate the accuracy and applicability of the model. In addition, the optimised friction materials should be evaluated in real-world operating environments to ensure their reliability and performance under dynamic conditions, thus bridging the gap between laboratory results and practical applications.

## Figures and Tables

**Table 1:** The chemical compositions of the two friction materials according to desired COF and temperature values.

Chemical Composition	First Material	Second Material
C	54.37	42.40
O	26.96	27.14
Al	1.72	1.92
Si	6.33	6.26
S	1.10	2.05
Ca	3.40	5.01
Fe	3.66	4.38
Cu	1.06	4.05
Ba	0.55	1.58
Zn	0.17	2.44
Ti	0.05	0.12
Na	0.10	0.75
K	0.40	0.12
Mg	0.14	-
Sn	-	0.99
P	-	0.43
Mn	-	0.40
Max COF values	-	0.47
Min temperature variation	0.1	

## Data Availability

The original contributions presented in this study are included in the article. Further inquiries can be directed to the corresponding author.
